# How are hospitals in England caring for women at risk of preterm birth in 2021? The influence of national guidance on preterm birth care in England: a national questionnaire

**DOI:** 10.1186/s12884-023-05388-w

**Published:** 2023-01-20

**Authors:** Naomi Carlisle, Angharad Care, Dilly O. C. Anumba, Sonia Dalkin, Jane Sandall, Andrew H. Shennan

**Affiliations:** 1 Department of Women and Children’s Health, The School of Life Course & Population Sciences, King’s College London, 10th Floor North Wing, St Thomas’ Hospital, Westminster Bridge Road, London, SE1 7EH UK; 2 Centre for Women and Children’s Health Research, University of Liverpool, Liverpool Women’s Hospital, Liverpool, UK; 3 Academic Unit of Reproductive and Developmental Medicine, Department of Oncology and Metabolism, University of Sheffield, Sheffield, UK; 4 Faculty of Health and Life Sciences, Northumbria University, Newcastle Upon Tyne, UK

**Keywords:** Preterm, Saving Babies Lives Care Bundle Version 2, Preterm pathway, Preterm clinic

## Abstract

**Background:**

National guidance (Saving Babies Lives Care Bundle Version 2 (SBLCBv2) Element 5) was published in 2019, with the aim to standardise preterm care in England. We plan to identify how many preterm birth surveillance clinics there are in England, and to define current national management in caring for women who are both asymptomatic and high-risk of preterm birth, and who arrive symptomatically in threatened preterm labour, to assist preterm management both nationally and internationally.

**Methods:**

An online survey comprising of 27 questions was sent to all maternity units in England between February 2021 to July 2021.

**Results:**

Data was obtained from 96 units. Quantitative analysis and free text analysis was then undertaken. We identified 78 preterm birth surveillance clinics in England, an increase from 30 preterm clinics in 2017. This is a staggering 160% increase in 4 years. SBLCBv2 has had a considerable impact in increasing preterm birth surveillance clinic services, with the majority (61%) of sites reporting that the NHS England publication influenced their unit in setting up their clinic. Variations exist at every step of the preterm pathway, such as deciding which risk factors warrant referral, distinguishing within particular risk factors, and offering screening tests and treatment options.

**Conclusions:**

While variations in care still do persist, hospitals have done well to increase preterm surveillance clinics, under the difficult circumstances of the COVID pandemic and many without specific additional funding.

**Supplementary Information:**

The online version contains supplementary material available at 10.1186/s12884-023-05388-w.

## Background

Around 40% of neonatal deaths globally are due to prematurity (birth before 37 weeks’ gestation) [1]. In 2020, 7.4% of babies were born preterm in England and Wales [2]. Over a decade ago, the cost of preterm birth was estimated as costing the National Health Service (NHS) £1 billion pounds annually [3].

The Department of Health is understandably keen to reduce the preterm birth rate in the UK to 6% by 2025 [4], which has been reiterated in The NHS Long Term Plan [5]. In March 2019, NHS England published Saving Babies Lives Care Bundle Version 2 (SBLCBv2) [6]. This updated version contained a new fifth element on recommendations for reducing preterm births. To standardise care, these recommendations were incorporated into the NHS standard contract for 2019/20 [7]. This is the first-time national guidance has formally mentioned specialist preterm birth surveillance clinics.

Previous surveys examining preterm birth surveillance and prevention practice in the United Kingdom have shown wide variations of care [8, 9]. The survey undertaken in 2017 [9] found a 30% increase in specialist clinics in England (from 23 to 30) compared to 5 years earlier [8]. Now that NHS England recommends that all women at high or intermediate risk of preterm birth are referred to a preterm birth surveillance clinic, we anticipate that clinics will increase further again in England.

A more focussed survey to evaluate the impact of the new guidelines, SBLCBv2, was therefore performed. We plan to identify how many preterm birth surveillance clinics there are in England, and to define current national management in caring for women who are both asymptomatic and high-risk of preterm birth and who arrive symptomatically in threatened preterm labour, to assist preterm management both nationally and internationally.

## Methods

An online survey comprising of 27 questions (see Supporting Information Additional file 1: Appendix S1) was developed utilising the secure, GDPR compliant, JISC online surveys platform. The survey was created by the authors, based upon previous surveys undertaken, and contained a mix of multiple choice and free text questions.

Since the 2017 questionnaire, some NHS trusts in England have been renamed, merged or dissolved. The link to the online survey was therefore sent to 126 trusts that provide maternity care in England, of which details were found from cross referencing the NHS Maternity Statistics for England 2019–2020 [10] with a list of NHS Acute Trusts [11]. These 126 trusts cover 187 units (155 units that provide obstetric-led intrapartum care, and 32 units or birth centres that only provide midwifery-led intrapartum care and/or antenatal/community care).

The survey was initially sent out to obstetricians and/or senior/specialist midwives in late February 2021. Sites that had not responded were contacted a maximum of 2 further times over the next few months until July 2021. The survey was closed to new responses in August 2021. Methods were carried out in accordance with the appropriate approvals and registration (King’s College London Research Governance Office (DPRF-20/21–17283)).

The data was downloaded onto an Excel spreadsheet. Free text questions where numerical answers were given were standardised. If a site gave a range of numbers as an answer, then the mean of this range was taken to produce a single number. In cases where the mean would be inappropriate then the number was rounded up (for example if the respondent wrote 1–2 women per year, this would be corrected to 2 women as 1.5 is imprecise). Two sites did not provide their birth rate, so this information was entered by the research team using information taken from CQC reports.

The project was registered with King’s College London Research Governance Office (DPRF-20/21–17,283).

### Funding

This project was undertaken as part of the IMPART study (IMplementation of the Preterm Birth Surveillance PAthway: a RealisT evaluation including a realist literature scope, ISRCTN57127874) [12] which is funded through a NIHR Clinical Doctoral Research Fellowship (NIHR300484) awarded to NC. The funder played no role in collecting or analysing the data, or writing the paper.

## Results

After removing blank responses (*n* = 2), and duplicate responses, (*n* = 6), data was obtained from 96 units (94 units that offer obstetric-led intrapartum care, and 2 units that provides maternity-led intrapartum care and/or antenatal/community care). This achieved an overall response rate of 51% (96/187), with a response rate of 61% (94/155) from obstetric led intrapartum units, which is similar to the 2017 questionnaire response rate.

### Caring for asymptomatic women at high risk of preterm birth

Overall, 78/96 units (81%) reported that they had a preterm birth surveillance clinic (see Table 1).

**Table 1:** Antenatal preterm birth surveillance provision

**Antenatal preterm birth surveillance provision**	n	%
YES – have a preterm birth surveillance clinic	78	81
OTHER—in the process of setting a clinic up	4	4
NO – appropriate women have consultant antenatal care	12	13
NO – appropriate women are referred to another hospital	2	2
TOTAL	96	100

A diverse range of sites responded to the questionnaire, representative of hospitals in England. The delivery rate of sites ranged from 1,400 births to 10,000 births per year (a mean of 4,196 births per year, and a median of 4,150 births per year). Overall, 28% (27/96) have a Level 1/ Special Care Baby Unit (for babies born over 32 weeks’ gestation), 38% (36/96) have a Level 2/ Local Neonatal Unit (for babies born 28–32 weeks’ gestation) and 34% (33/96) have a Level 3/ Neonatal Intensive Care Unit (for babies born before 28 weeks’ gestation). The two units who referred women to another hospital for preterm surveillance care antenatally, have less than 2,800 births per year and both have Level 1/ Special Care Baby Units.

Most preterm birth surveillance clinics are run weekly (82%), fully funded by the NHS (91%) and led by an NHS consultant who is principally obstetrics based (82%) (see Table 2).

**Table 2:** Frequency, clinical lead, and funding of preterm surveillance clinics

	**Frequency, clinical lead, and funding of preterm birth surveillance clinics**	n	%
How frequently do you run the clinic?	Other—Twice weekly	2	3
	Weekly	63	82
	Fortnightly	11	14
	Monthly	1	1
	TOTAL	77	100
Who is the clinical lead for this clinic?	NHS Consultant (principally gynaecology)	4	5
	NHS Consultant (principally obstetrics)	63	82
	Other—Two consultants jointly lead (one employed by the NHS, one employed with a university)	3	4
	Subspecialist	4	5
	University staff clinician	3	4
	Specialist trainee doctor	0	0
	Midwife	0	0
	TOTAL	77	100
How is your clinic funded?	NHS	68	91
	Research	3	4
	Joint NHS and research	1	1
	Don’t know	3	4
	TOTAL	75	100

### Indications for referral to a preterm surveillance clinic

A full list of indications for referral to a specialist preterm labour clinics are highlighted in Table 3.

**Table 3:** Indication for referral to preterm surveillance clinic

**Indication for referral to preterm surveillance clinic (non-exclusive)**	n	%
Previous Spontaneous Preterm Birth/ mid-trimester loss	86	100
Previous preterm prelabour rupture of membranes (PPROM)	83	97
Previous cervical cerclage	82	95
Uterine variant (i.e. unicornuate, bicornuate uterus or uterine septum)	82	95
Intrauterine adhesions (Ashermann’s syndrome)	57	66
History of trachelectomy (for cervical cancer)	82	95
Previous delivery by caesarean section in labour	53	62
Cervical excisional event -Single LLETZ (any depth removed)	20	23
Cervical excisional event -Single LLETZ (more than 10 mm removed only)	68	79
Other—Cervical excisional event -Single LLETZ (more than 20 mm removed only)	1	1
Cervical surgery—Multiple LLETZ or cone biopsy	84	98
Recurrent first trimester miscarriage	8	9
Following episode of threatened preterm labour	16	19
Incidental finding of short cervix without preterm birth history	71	83
Other—Connective tissue disorder	2	2
TOTAL	86	100

From our respondents, only one common risk factor was noted as being acceptable for referral in every clinic in England- which was having a previous spontaneous preterm birth or mid-trimester loss. However, the gestational cut off for referrals could vary between the sites.

Of the 62% of clinics who accept referrals from women who had a previous delivery by caesarean section in labour, the majority (90%, 46/51) accept women who had a previous delivery by caesarean section at full (10 cm) cervical dilatation (see Table 1 in Supporting Information Additional file 2: Appendix S2). Full results regarding indications for referral to preterm birth surveillance clinics can be seen in Supporting Information Additional file 2: Appendix S2.

Majority of sites (61%, 47/77) reported that SBLCBv2 influenced their unit in setting up their preterm birth surveillance clinic. Meanwhile 38% (29/77) said SBLCBv2 did not influence their unit’s clinic, and one site said they did not know. Most sites (65%, 55/85), felt their referral criteria had changed because of SBLCBv2, 34% of sites (29/85) said their referral criteria had not changed as a result SBLCBv2, and one site did not know. Of the sites that gave their clinic’s referral criteria, 36% (31/86) included all the risk factors that are outlined in the SBLCBv2 ‘’suggested risk assessment’’ for preterm birth [6].

### Treatment offered at a preterm birth surveillance clinic

Nearly half of sites (47%, 40/86) reported that they sometimes offered asymptomatic women a prophylactic vaginal cerclage on history alone (see Table 4). Of the 40 free text comments associated with this answer, 95% (38/40) commented that this depended on the woman’s history. Some gave specific criteria which varied between ‘one mid-trimester loss’ to ‘three mid-trimester losses.’ Other sites described their policy as ‘a few’ or ‘multiple’ mid trimester losses. A quarter of the comments (10/40) reported that offering this depended on the woman’s wishes.

**Table 4:** Treatment offered at a preterm surveillance clinic

	**Asymptomatic treatment offered in preterm surveillance clinics**	n	%
For asymptomatic women at risk of preterm labour, do you offer prophylactic vaginal cerclage on history alone without surveillance with ultrasound?	Yes	17	20
	No	29	34
	Some	40	47
	TOTAL	86	100
For asymptomatic women at risk of preterm labour, do you offer prophylactic vaginal progesterone on history alone without surveillance with ultrasound?	Yes	14	16
	No	47	55
	Some	25	29
	TOTAL	86	100
What is your preferred primary treatment for short cervical length?	Vaginal progesterone	16	19
	Cervical cerclage (Braided suture)	38	44
	Cervical cerclage (Monofilament suture)	16	19
	Vaginal pessary (such as Arabin)	0	0
	IM Progesterone	0	0
	Combination	19	22
	Other	11	13
	TOTAL	86	100

Full results regarding referrals to and from preterm birth surveillance clinics, screening currently offered, and treatment offered at preterm birth surveillance clinics can be seen in Supporting Information Additional file 2: Appendix S2.

### Implementing a preterm birth surveillance clinic

Seventy-three sites that responded left a free text comment on what helped implement their preterm birth surveillance clinic. A third, (33%, 24/73) felt that SBLCBv2 helped. Some, 22% (16/73) felt that having someone motivated (either a midwife or obstetrician) helped. Some, 14% (10/73) noted external support (either via individuals, particular hospitals or through regional networks). Of those that stated this, 3 sites mentioned St Thomas’ Hospital, London specifically, 3 sites mentioned emailing NC (author), 1 site mentioned Liverpool Women’s Hospital and 1 site mentioned Leeds Teaching Hospitals NHS Trust. Eight sites, (11%) felt nothing particularly helped. Four sites (5%) highlighted the aid of additional funding. The form of this funding varied (in one site it enabled employing a specialist midwife, another it allowed admin time, in another NHS Innovation funding enabled fetal fibronectin purchasing, and another received Local Maternity System funding to support the service for 12 months before NHS trust funding was agreed). Four sites felt strong managerial support enabled them to successfully set up their clinic, and 2 sites felt that good practical facilities (such as easy access to a fetal fibronectin machine) helped.

Seventy-two sites left free text comments regarding what hindered implementation of their clinic. Over half of respondents (56%, 40/72) highlighted capacity issues. These ranged from too many referrals to cope with, a lack of appropriate clinical space, a lack of staff, and/or a lack of resources. This included 19% (14/72) who specifically mentioned a lack of clinicians able to scan and/or lack of scanning equipment. Several sites (10%, 7/72) required a clinic midwife. Some (4%, 3/72) highlighted absent inclusion in job roles and 3% (2/72) mentioned delays in business case approvals. This leads to sites unable to implement ‘in full accordance with SBLCBv2’. Three sites felt their small numbers of appropriate women created difficulty in assigning a clinical lead, and pragmatically included these women in their general obstetric antenatal clinic. COVID was mentioned (6%, 4/72) as hindering implementation. One site felt ambiguity on how sites should care for these women, partly due to guidance overload. Meanwhile 29% (21/72) felt nothing hindered set up.

Full results regarding the impact of preterm birth surveillance clinics and caring for women who arrive in threatened preterm labour, can be seen in Supporting Information Additional file 2: Appendix S2.

## Discussion

### Main findings

We have now identified 78 preterm birth surveillance clinics in England, an increase from 30 preterm clinics in 2017 [9]. This is a staggering 160% increase in 4 years.

SBLCBv2 has had a considerable impact in increasing preterm birth surveillance clinic services, with the majority (61%) of sites reporting that the NHS England publication influenced their unit in setting up their clinic. Most sites, 65%, also felt that their referral criteria had changed because of SBLCBv2.

### Strengths and limitations

The overall response rate was 51%, with a response rate of 61% from obstetric led intrapartum units. We feel we achieved effective geographical spread and representation from sites across England, including a range of units with different delivery rates, and units with a variety of neonatal levels. These results could assist preterm management both nationally and internationally.

Often units that provide obstetric-led intrapartum care manage higher risk pregnancies antenatally and are more likely to have antenatal preterm birth services. This may be one reason why we did not receive a high response from units that provide maternity-led intrapartum care and/or antenatal/community care. However, it was important that the questionnaire was sent to individual units, not hospital trusts. This meant we could understand how units work both individually and how they interact (e.g., through estimated referral numbers) with other units in their local area.

However, referrals from other units did not include women who purposefully booked their pregnancy at a unit with a good reputation for preterm birth surveillance care. The continuing expansion of online support groups and awareness of risk factors means we cannot underestimate the impact of women rightfully influencing their own care in this way, especially in densely populated cities where a variety of hospitals can easily be accessed.

### Interpretation

While SBLCBv2 has succeeded in promoting preterm surveillance care and clinics, it has not achieved standardised care across England. Variations in practice still exist at every step of the preterm pathway, such as deciding which risk factors warrant referral, distinguishing within particular risk factors, and offering screening tests and treatment options. The 2017 questionnaire found three common risk factors across all clinics (previous spontaneous preterm birth, previous 2 × LLETZ procedures, and previous cone biopsy). This questionnaire found only one common risk factor across all clinics (which was having a previous spontaneous preterm birth or mid-trimester loss).

This could be due to different reasons, one of which being the non-prescriptive nature of SBLCBv2. For example, SBLCBv2 provides a *suggested* risk assessment, which 36% of clinics seem to be following. However, SBLCBv2 also expressed that alternative risk assessments that have been agreed with site’s local commissioners could be equally appropriate meaning remaining sites may be working under an agreed alternative.

Variations in care could be due to capacity issues, which was highlighted by 56% of respondents as hindering implementation. This has led to some operating a preterm surveillance clinic with a slimmed-down referral criteria that do not cover all the risk factors mentioned in SBLCBv2.

A lack of, or unclear, evidence bases, could also lead to these variations of care which was highlighted by respondents. The C-STITCH study [13] results will hopefully provide more clarity on whether clinicians should favour monofilament or braided cervical sutures. Studies comparing cerclage, progesterone and Arabin pessaries [14, 15] could also help to provide clearer treatment pathways for a short cervix. This could be compounded by the fast pace of clinical evidence being published in this area, sometimes with conflicting results. For example, only recently was the EPPPIC meta-analysis published [16], which alongside other reviews [17] has given the field a consensus on progesterone’s role in preventing preterm birth after previous contradictory research conclusions.

The questionnaire demonstrated the pragmatic capability of sites in setting up new preterm birth surveillance pathways. Preterm surveillance can be just as effective situated within an antenatal clinic, rather than a standalone specific preterm surveillance clinic – which sites with smaller numbers noted in the free text comments. Nearly a fifth (14%) noted how collegial external support helped them implement their clinic, highlighting the importance of networking between local sites and more experienced sites being open to sharing their resources such as protocols and guidelines.

Biomarker tests, specifically quantitative fetal fibronectin, are the most popular tool for assessing symptomatic women in threatened preterm labour. One site did highlight how they did not have 24/7 access to cervical length scanning, which could explain why only 38% of sites routinely undertake cervical length scans on women arrive in threatened preterm labour. While training clinicians to scan is time consuming, individual cervical length scans are inexpensive compared to biomarker tests.

Nearly half (49%) of sites are using the QUiPP App in asymptomatic women, and just over half (51%) in symptomatic women where it is recommended in the majority (78%) of local guidelines. Its popularity may have been boosted by the recent EQUIPTT study [18–20] and the free to access QUiPP App Toolkit [21], which was recommended in the SBLCBv2 COVID-19 update Appendix I [22].

## Conclusion

Hospital sites in England have done well to increase preterm surveillance clinics, under the difficult circumstances of the COVID pandemic and many without specific additional funding.

Wide variations of preterm care, some at variance with guidelines and the evidence base, in both asymptomatic and symptomatic women, exists. Increased collaboration between sites will hopefully result in more homogenous care.

Nearly all clinics (91%) are funded by the NHS, demonstrating the readiness to have this incorporated into mainstream care. Future work (such as the IMPART study [12]), will understand how best to support successful preterm pathway implementation.

## Supplementary Information

**Additional file 1: Appendix S1.** 

**Additional file 2: Appendix S2.** 

## Data Availability

The datasets generated and/or analysed during the current study are not publicly available as they containing information that could compromise the privacy of individual sites, but are available from the corresponding author on reasonable request.

## Abbreviations

CQC: Care Quality Commission
GDPR: General Data Protection Regulation
NHS: National Health Service
SBLCBv2: Saving Babies Lives Care Bundle Version 2

## References

[1] WHO (2015). Born Too Soon: Preterm Birth In Europe. Trends, Causes and Prevention.

[2] ONS (2022). Birth characteristics in England and Wales: 2020.

[3] Mangham LJ, Petrou S, Doyle LW, Draper ES, Marlow N (2009). The cost of preterm birth throughout childhood in England and Wales. Pediatrics.

[4] Department of Health. Safer Maternity Care - The National Maternity Safety Strategy - Progress and Next Steps. 2017. Available from: http://www.euro.who.int/__data/assets/pdf_file/0004/277735/Born-too-soon_preterm-birth-in-Europe-trends,-causes-and-prevention.pdf?ua=1

[5] NHS England. z NHS Long Term Plan » Overview and summary. NHS England; 2019. Available from: https://www.longtermplan.nhs.uk/online-version/overview-and-summary/

[6] NHS England. a Saving Babies Lives Care Bundle Version 2. 2019. Available from: https://www.england.nhs.uk/wp-content/uploads/2019/03/Saving-Babies-Lives-Care-Bundle-Version-Two-Final-Version2.pdf

[7] NHS England (2019). NHS Standard Contract 2019/20.

[8] Sharp AN, Alfirevic Z (2014). Provision and practice of specialist preterm labour clinics: a UK survey of practice. BJOG.

[9] Care A, Ingleby L, Alfirevic Z, Sharp A. The influence of the introduction of national guidelines on preterm birth prevention practice: UK experience. BJOG. 2019;0(0). Available from: 10.1111/1471-0528.15549 10.1111/1471-0528.15549 PMC6590292 30461172

[10] NHS Digital (2020). NHS Maternity Statistics, England 2019–20.

[11] NHS Website (2017). Acute Trust List.

[12] Carlisle N, Dalkin SM, Shennan AH, Sandall J (2022). Protocol for the IMPART study: IMplementation of the preterm birth surveillance PAthway – a RealisT evaluation. BMJ Open.

[13] Israfil-Bayli F, Morton VH, Hewitt CA, Ewer AK, Gray J, Norman J (2021). C-STICH: Cerclage Suture Type for an Insufficient Cervix and its effect on Health outcomes—a multicentre randomised controlled trial. Trials.

[14] Care A, Jackson R, O’Brien E, Leigh S, Cornforth C, Haycox A (2021). Cervical cerclage, pessary, or vaginal progesterone in high-risk pregnant women with short cervix: a randomized feasibility study. J Matern Fetal Neonatal Med.

[15] Hezelgrave NL, Watson HA, Ridout A, Diab F, Seed PT, Chin-Smith E (2016). Rationale and design of SuPPoRT: a multi-centre randomised controlled trial to compare three treatments: cervical cerclage, cervical pessary and vaginal progesterone, for the prevention of preterm birth in women who develop a short cervix. BMC Pregnancy Childbirth.

[16] Stewart LA, Simmonds M, Duley L, Llewellyn A, Sharif S, Walker RAE (2021). Evaluating Progestogens for Preventing Preterm birth International Collaborative (EPPPIC): meta-analysis of individual participant data from randomised controlled trials. The Lancet.

[17] Care A (2022). Interventions to prevent spontaneous preterm birth in high-risk women with singleton pregnancy: a systematic review and network meta-analysis. BMJ.

[18] Watson HA, Carlisle N, Kuhrt K, Tribe RM, Carter J, Seed P (2019). EQUIPTT: The Evaluation of the QUiPP app for Triage and Transfer protocol for a cluster randomised trial to evaluate the impact of the QUiPP app on inappropriate management for threatened preterm labour. BMC Pregnancy Childbirth.

[19] Carlisle N, Watson HA, Seed PT, Carter J, Kuhrt K, Tribe RM (2021). Impact of a medical mobile phone app (QUiPP) for predicting preterm birth on the anxiety and decisional conflicts faced by women in threatened preterm labour. Midwifery.

[20] Carlisle N, Watson HA, Kuhrt K, Carter J, Seed PT, Tribe RM (2021). Ten women’s decision-making experiences in threatened preterm labour: qualitative findings from the EQUIPTT trial. Sex Reprod Healthc..

[21] Carlisle N, Watson HA, Shennan AH (2021). Development and rapid rollout of The QUiPP App Toolkit for women who arrive in threatened preterm labour. BMJ Open Qual.

[22] NHS England (2020). Appendix I: Implications of COVID-19 on reducing preterm births.

[23] Twycross A, Shorten A (2014). Service evaluation, audit and research: what is the difference?. Evid Based Nurs.

