# Supplementary material for: How are hospitals in England caring for women at risk of preterm birth in 2021? The influence of national guidance on preterm birth care in England: a national questionnaire
Source: BMC Pregnancy Childbirth. 2023 Jan 20;23:47. doi: 10.1186/s12884-023-05388-w (PMC9854090; doi:10.1186/s12884-023-05388-w)
Supplement: Supplementary file 1 — Additional file 1: Appendix S1. [file 12884_2023_5388_MOESM1_ESM.pdf]

Survey of Preterm Birth Practice in the United Kingdom

Page 1

Background: Our previous survey of practice (Care *et al.*, 2019) found that UK Preterm Birth Prevention Clinics had increased by 44% over 5 years. Since then, NHS England has recommended that all hospitals in England have a pathway in place for women with antenatal risk factors for preterm birth (NHS England, 2019a) with an aim of reducing the preterm birth rate from 8% to 6% by 2025 (Department of Health, 2017; NHS England, 2019b). In light of this, we wish to review how the practise and provision of care for women at risk of preterm birth has changed over time.

Aim: To identify the current state of preterm birth practice within the UK

Please tick the response that best represents your department's policy.

If answering other, please specify how your management differs from the written responses.

If you need to contact us about this privacy notice, please contact naomi.h.carlisle@kcl.ac.uk

Why do we process your personal information? We process your name and email in order to contact you if we require further information or clarity on your answers. We process your hospital site to ensure we do not analyse duplicate responses from the same hospital.

What is our 'legal basis for processing'? Data protection legislation allows us to use your personal information in this way because you have actively given us your permission by completing this questionnaire. You may withdraw your consent at any time by emailing naomi.h.carlisle@kcl.ac.uk

Do I have to provide personal information? No – if you don't it just means we will not have preterm information from your hospital site.

How long do you keep the information for? We will keep your information until we have closed the questionnaire and analysed all information provided.

Do you share information with other organisations? No - they will only be seen by Naomi Carlisle. All information will be deleted after analysis.

To find out more about how the university deals with your personal information, including your rights and who to contact if you have a concern, please see the university's core privacy notice at https://www.kcl.ac.uk/terms/privacy.aspx

What is your name?

[ ]

What is your email address?

[ ]

What hospital do you work at?

- ○ Airedale General Hospital
- ○ Alnwick Infirmary (Birth centre)
- ○ Andover War Memorial Hospital
- ○ Arrowe Park Hospital
- ○ Barkantine Birth Centre

- Barking Community Birth Centre
- Barnet Hospital
- Barnsley Hospital
- Basildon University Hospital
- Basingstoke and North Hampshire Hospital
- Bassetlaw Hospital
- Bedford Hospital South Wing
- Berwick Materity Unit
- Birmingham City Hospital
- Birmingham Womens Hospital
- Blackpool Victoria Hospital
- Bradford Royal Infirmary
- Broomfield Hospital
- Burnley General Hospital
- Calderdale Royal Hospital
- Chelsea and Westminster Hospital
- Cheltenham General Hospital (Aveta Birth Centre)
- Chesterfield Royal Hospital NHS Foundation Trust
- Chorley and South Ribble Hospital
- Clacton and District Hospital
- Colchester Hospital
- Conquest Hospital
- Cossham Birth Centre at Cossham Hospital
- Countess of Chester Hospital
- Crowborough Birth Centre
- Croydon University Hospital
- Cumberland Infirmary
- Darent Valley Hospital
- Darlington Memorial Hospital
- Derriford Hospital
- Dewsbury and Distrct Hospital (Bronte Birth Centre)

- Diana, Princess of Wales Hospital, Grimsby
- Doncaster Women's Hospital
- Dorset County Hospital
- East Surrey Hospital
- Eastbourne District General Hospital
- Epsom Hospital
- Friarage Hospital
- Frimley Park Hospital
- Furness General Hospital
- George Eliot Hospital
- Gloucester Royal Hospital
- Good Hope
- Goole and District Hospital
- Gosport War Memorial Hospital (Blake materity care)
- Great Western Hospital
- Harrogate District Hospital
- Heartlands Hospital
- Hexham General Hospital (birth centre)
- Hinchingbrooke Hospital
- Homerton Row
- Horton General Midwifery unit
- Huddersfield Royal Infirmary
- Hull Royal Infirmary
- Ipswich Hospital
- James Cook University Hospital
- James Paget University Hospitals NHS Foundation Trust
- Jessop Wing, Sheffield
- John Radcliffe Hospital, The Women's Centre
- Kettering General Hospital
- King George Hospital, Ilford
- King's College Hospital

- King's Mill Hospital
- Kingston Hospital NHS Foundation Trust
- Leeds General Infirmary
- Leicester General Hospital
- Leicester Royal Infirmary
- Leighton Hospital
- Lincoln County Hospital
- Lister Hospital
- Liverpool Women's NHS Foundation Trust
- Luton and Dunstable University Hospital
- Macclesfield District General Hospital
- Maidstone Birth Centre
- Manor Hospital, Walsall
- Medway Maritime Hospital
- Milton Keynes Hospital
- Musgrove Park Hospital
- New Cross Hospital
- Newham University Hospital
- Norfolk and Norwich University Hospital
- North Devon District Hospital
- North Manchester General Hospital
- North Middlesex University Hospital
- Northampton General Hospital
- Northumbria Specialist Emergency Care Hospital
- Northwick Park Hospital
- Nottingham City Hospital
- Ormskirk District General Hospital
- Penrith Birthing Centre at Penrith Community Hopsital
- Peterborough City Hospital
- Pilgrim Hospital
- Pinderfields Hospital

- Pontefract Hospital (Friarwood Birth Centre)
- Poole Hospital
- Wansbeck General Hospital
- University Hospital Southampton
- Princess Royal Hospital, Haywards Heath
- Princess Royal Hospital, Shropshire
- Princess Royal University Hospital, Orpington
- Queen Alexandra Hopsital, Portsmouth
- Queen Charlotte's & Chelsea Hospital
- Queen Elizabeth Hospital, Gateshead
- Queen Elizabeth Hospital, Woolwich
- Queen Elizabeth I I Hospital, Welwyn Garden City
- Queen Elizabeth The Queen Mother Hospital, Margate
- Queen's Hospital, Burton-on-Trent, Staffordshire
- Queen's Hospital, Romford
- Queen's Medical Centre, Nottingham
- Rotherham Hospital
- Royal Albert Edward Infirmary, Wigan
- Royal Berkshire Hospital
- Royal Blackburn Hospital
- Royal Bolton Hospital
- Royal Bournemouth Hospital
- Wansbeck General Hospital
- Royal Cornwall Hospital
- Royal Derby Hospital
- Royal Devon and Exeter Hospital (Wonford Hospital)
- Royal Free Hospital
- Royal Hampshire County Hospital
- Royal Lancaster Infirmary
- Royal Preston Hospital
- Royal Stoke University Hospital

- Royal Surrey County Hospital
- Royal Sussex County Hospital
- Royal United Hospitals, Bath
- Royal Victoria Infirmary, Newcastle upon Tyne
- Russells Hall Hospital
- Salford Royal NHS Foundation Trust
- Salisbury District Hospital
- Samuel Johnson Community Hospital, Lichfield
- Scarborough Hospital
- Scunthorpe General Hospital
- Solihull Hospital
- South Tyneside District HospitalHarton Lane
- Southend University Hospital
- Southmead Hospital
- Southport Hospital
- St George's University Hospitals NHS Foundation Trust
- St Helier Hospital
- St James's University Hospital, Leeds
- St Mary's Hospital, Leicestershire
- St Mary's Hospital, London
- St Mary's Hospital,Manchester
- St Michael's Hospital, Bristol
- St Peter's Hospital, Surrey
- St Richard's Hospital, West Sussex
- St Thomas' Hospital
- St. Mary's Hospital, Isle of Wight
- Stepping Hill Hospital
- Stoke Mandeville Hospital
- Stroud Maternity Unit
- Sunderland Royal Hospital
- Tameside & Glossop Integrated Care NHS Foundation Trust

- The County Hospital, Wye Valley NHS Trust
- The Hillingdon Hospital
- The Princess Alexandra Hospital NHS Trust, Harlow Essex
- The Queen Elizabeth Hospital, King's Lynn Norfolk
- The Rosie Hospital, Cambridge
- The Royal London Hospital
- The Royal Oldham Hospital
- The Whittington Hospital
- The York Hospital
- Tunbridge Wells Hospital
- Torbay Hospital
- University College Hospital London
- University Hospital Lewisham
- University Hospital Of Hartlepool
- University Hospital of North Durham
- University Hospital of North Tees
- University Hospital, Coventry
- Warrington Hospital
- Warwick Hospital
- Watford General Hospital
- West Cumberland Hospital
- West Middlesex University Hospital
- West Suffolk Hospital
- Westmoreland General
- Wexham Park Hospital
- Whipps Cross University Hospital
- Whiston Hospital
- Whitelake Midwifery Led Unit (Newton Abbot Community Hospital)
- William Harvey Hospital
- Worcestershire Royal Hospital
- Worthing Hospital

- Wycombe Hospital Birth Centre
- Wythenshawe Hospital
- Yeovil District Hospital
- Other

If you selected Other, please specify:

Approximately how many deliveries does your unit have per year?

What neonatal care does your unit provide?

- Level 1/ Special Care Baby Unit (babies born over 32 weeks' gestation)
- Level 2/ Local Neonatal Unit (babies born 28-32 weeks')
- Level 3/ Neonatal Intensive Care Unit (babies born before 28 weeks'

Does your unit routinely scan for a short cervix in women who are expecting twins?

- Yes - All twins
- Yes - Some twins
- Yes - Scanned in another clinic
- No
- Don't know

Do you have a preterm labour (PTL) clinic for women with antenatal risk factors for preterm birth?

- YES
- NO – they receive consultant antenatal care
- NO – they are referred to another hospital
- Other

If you selected Other, please specify:

Asymptomatic high risk women questions

Did the Saving Babies Live’s Care Bundle V2 publication influence your unit setting up the PTL clinic?

- No
- Yes
- Don’t know

How frequently do you run this clinic?

- Weekly
- Fortnightly
- Monthly
- Other

If you selected Other, please specify:

Please estimate how many new referrals your PTL clinic receives a year?

Who is the clinical lead for this clinic?

- NHS consultant (Principally Obstetrics)
- NHS consultant (Principally Gynaecology)
- Subspecialist
- University Staff Clinician

- Specialty Trainee Doctor
- Midwife
- Other

If you selected Other, please specify:

Are you aware how your clinic is funded?

- NHS
- Research
- Don’t know
- Other

If you selected Other, please specify:

Your PTL clinic generally sees the highest risk women for appointments from around ______ weeks to ______ weeks gestation (write down your lower and upper limits in weeks please)

Asymptomatic high-risk women questions.

What is your referral criteria? Do you see patients with a history of… (select all that apply)

- [ ] Previous Spontaneous Preterm Birth/ mid-trimester loss
- [ ] Previous preterm prelabour rupture of membranes (PPROM)
- [ ] Previous cervical cerclage
- [ ] Uterine variant (i.e. unicornuate, bicornuate uterus or uterine septum)
- [ ] Intrauterine adhesions (Ashermann's syndrome)
- [ ] History of trachelectomy (for cervical cancer)
- [ ] Previous delivery by caesarean section in labour
- [ ] Cervical excisional event – Single LLETZ (any depth removed)
- [ ] Cervical excisional event – Single LLETZ (more than 10mm removed only)
- [ ] Cervical surgery – Multiple LLETZ or cone biopsy
- [ ] Recurrent first trimester miscarriage
- [ ] Following episode of threatened preterm labour
- [ ] Incidental finding of short cervix without preterm birth history
- [ ] Other

If you selected Other, please specify:

____

If you selected 'previous Spontaneous Preterm Birth/ mid-trimester loss' - From what gestation? ______weeks to______weeks

____

If you selected 'previous preterm prelabour rupture of membranes (PPROM)' - From what gestation?

If you selected 'previous delivery by caesarean section in labour' - From what dilatation? _____cm to_____cm

If you selected 'recurrent first trimester miscarriage' - How many?

Has your referral criteria changed as a result of Saving Babies Lives Care Bundle V2?

- ○ No
- ○ Yes
- ○ Don’t know

What screening tests do you offer asymptomatic women at risk of preterm birth? (Please tick all that apply)

- ☐ Transvaginal cervical length scan
- ☐ Qualitative fetal fibronectin
- ☐ Quantitative fetal fibronectin
- ☐ Actim Partus
- ☐ Partosure
- ☐ QUiPP app
- ☐ Urinalysis
- ☐ High vaginal swab

- [ ] Low vaginal swab
- [ ] Endocervical swab
- [ ] Other

If you selected Other, please specify:

If you undertake transvaginal cervical length scans, who performs them? (Tick all that apply)

- [ ] Question not applicable (do not undertake transvaginal cervical length)
- [ ] Consultant
- [ ] Specialty Trainee
- [ ] Non-training grade clinical staff
- [ ] Research Staff
- [ ] Midwife
- [ ] Ultrasonographer
- [ ] Other

If you selected Other, please specify:

For asymptomatic women at risk of preterm labour, do you offer prophylactic vaginal cerclage on history alone without surveillance with ultrasound?

- YES
- NO
- SOME

Please describe

For asymptomatic women at risk of preterm labour, do you offer prophylactic vaginal progesterone on history alone without surveillance with ultrasound?

- YES
- NO
- SOME

Please describe

What is your preferred primary treatment for short cervical length?

- Vaginal progesterone
- Cervical cerclage (Braided suture)
- Cervical cerclage (Monofilament suture)
- Vaginal pessary (such as Arabin)

- IM progesterone
- Combination treatment
- Other

If you selected Other, please specify:

Please state

If primary treatment for short cervical length fails, what is your choice of secondary treatment?

Asymptomatic questions

How many asymptomatic women would you estimate you refer to other units per year?

How many asymptomatic women would you estimate you receive from other units per year?

Symptomatic women questions

Moving on to women who arrive at your unit with symptoms of threatened preterm labour:

How do you assess symptomatic women at risk of preterm labour? (Please tick all that apply)

- ☐ Transvaginal cervical length scan
- ☐ Qualitative fetal fibronectin
- ☐ Quantitative fetal fibronectin
- ☐ Actim Partus
- ☐ Partosure
- ☐ QUiPP app
- ☐ Other

If you selected Other, please specify:

If you selected QUiPP App is this:

What criteria would you usually use to decide to transfer a woman to another unit?

- ◯ When her cervical length is less than_______mm
- ◯ Based upon biomarker test result

- Based upon the QUIPP app result
- Not sure
- Other

If you selected Other, please specify:

If selected 'when cervical length is less than__mm' - Please provide a value

If selected based upon biomarker test result

- Positive/negative result
- More than 50ng/ml (fetal fibronectin)
- More than 200 ng/mL (fetal fibronectin)
- Other

If you selected Other, please specify:

If selected based upon QUiPP App result

- When the risk of delivery within 1 week is 5% of more
- Other

If you selected Other, please specify:

For what indication would you offer tocoloysis?

- Transfer to another unit
- Steroids
- Don’t use
- Other
- Steroids AND transfer to another unit

If you selected Other, please specify:

At what gestation do you normally offer Steroids? (Please write lowest and latest gestation in weeks)

Do you offer ‘rescue’ cerclage for prolapsed membranes in asymptomatic women with no

contraindications?

- Yes
- No
- It depends on gestation
- Only to recruit to research/trial

Has/did anything in particular helped the set up/implementation of your PTL clinic?

Has/did anything hindered the set up/implementation of your PTL clinic?

Has providing your PTL clinic had a direct impact/effect on other parts of your service?

Thank you for your time in completing this questionnaire

Final page

Thanks for your time.

Any questions or further comments please email: naomi.h.carlisle@kcl.ac.uk

---

Key for selection options

20.b - If you selected QUiPP App is this:

Recommended in local guidance
Not recommended in local guidance but the majority use it
Not recommended in local guidance but some use it
