# Supplementary material for: How are hospitals in England caring for women at risk of preterm birth in 2021? The influence of national guidance on preterm birth care in England: a national questionnaire
Source: BMC Pregnancy Childbirth. 2023 Jan 20;23:47. doi: 10.1186/s12884-023-05388-w (PMC9854090; doi:10.1186/s12884-023-05388-w)
Supplement: Supplementary file 2 — Additional file 2: Appendix S2. [file 12884_2023_5388_MOESM2_ESM.docx]

# Supporting Information Appendix S2

## Indications for referral to a specialist preterm labour clinic

A full list of indications for referral to a specialist preterm labour clinics are highlighted in Table 3 in the main manuscript.

Most units, 81% (76/94), do not routinely scan for a short cervix in women who were expecting twins. Meanwhile 15% of units (14/94) scan ‘some women who were pregnant with twins’ and 4% (4/94) scan all women who are pregnant with twins.

The gestational range which clinics stated they see their highest risk women for appointments differed from 10-16 weeks’ gestation for their first appointment, to 14 weeks - term for when they are often discharged, in varying combinations. The most frequently noted gestation to initially see high-risk women was at 16 weeks’ gestation (25/76, 33%), and for them to then be discharged at 24 weeks’ gestation (24/74, 33%).

The gestation from which clinics accept referrals for a previous spontaneous preterm birth or mid-trimester loss ranged from their previous loss occurring at 12- 24 weeks’, to 22- 36 weeks’ gestation, in varying combinations. The most frequently noted referral gestation bounds were a loss that occurred between 16 weeks’ gestation (46/81, 57%) to 34 weeks’ gestation (52/83, 63%).

The gestation from which clinics accept referrals for a previous preterm prelabour rupture of membranes (PPROM) ranged from the PPROM occurring at 12- 24 weeks’, to 24 - 36 weeks’ gestation, in varying combinations. The most frequently noted referral gestation bounds were a PPROM that occurred between 16 weeks’ gestation (35/64, 55%) to 34 weeks’ gestation (42/62, 68%).

Of those 8 sites who see women who have a history of recurrent first trimester miscarriage, 88% (7/8) accept referrals after 3 recurrent miscarriages, while the remaining site described their referral policy as seeing women with ‘any’ recurrent miscarriage history.

The majority of sites who accept referrals from women who have had a previous delivery by caesarean section in labour, accept those where this occurred at 10cm dilated/fully dilated (90%, see Table 1 S2 below). Only 4% of sites accept women who were less than 9cm dilated when the caesarean section in labour occurred.

Table 1 S2 Dilation at previous caesarean delivery to be accepted for referral to preterm clinic

| **If 'previous delivery by caesarean section in labour' was selected as a referral criterion -from what dilatation?** | n | % |
| --- | --- | --- |
| 0 -10 cm | 1 | 2 |
| 4 -10cm | 1 | 2 |
| 9 -10 cm | 3 | 6 |
| 10cm only | 46 | 90 |
| TOTAL | 51 | 100 |

## Referrals to and from preterm birth surveillance clinics

Two sites report referring 100-300 asymptomatic women per year to other units (see Table 2 S2 below). Both of these sites have their own preterm birth surveillance clinic, and are medium sized units (approximately 5,000 - 5,500 deliveries per year). The two sites that received 100-200 asymptomatic referrals from other units are both large (over 6,500 deliveries per year) tertiary units equipped with a Level 3/ Neonatal Intensive Care Unit.

Table 2 S2 Referrals for preterm surveillance care

|  | **Referrals for preterm surveillance care** | n | % |
| --- | --- | --- | --- |
| Please estimate how many new referrals your clinic receives a year | 0-49 | 2 | 4 |
|  | 50-99 | 6 | 11 |
|  | 100-149 | 10 | 18 |
|  | 150-199 | 11 | 19 |
|  | 200- 249 | 6 | 11 |
|  | 250-299 | 7 | 12 |
|  | 300-399 | 8 | 14 |
|  | 400-499 | 2 | 4 |
|  | 500- 599 | 2 | 4 |
|  | 650-699 | 0 | 0 |
|  | 700-799 | 0 | 0 |
|  | 800-899 | 1 | 2 |
|  | 900-999 | 1 | 2 |
|  | 1000- 1099 | 0 | 0 |
|  | 1100-1199 | 0 | 0 |
|  | 1200-1299 | 0 | 0 |
|  | 1300-1399 | 0 | 0 |
|  | 1400- 1499 | 0 | 0 |
|  | 1500-1599 | 1 | 2 |
|  | TOTAL | 57 | 100 |
|  |  |  |  |
| How many asymptomatic women would you estimate you refer to other units per year? | 0 | 38 | 50 |
|  | 1-9 | 26 | 34 |
|  | 10-19 | 7 | 9 |
|  | 20-49 | 1 | 1 |
|  | 50-99 | 2 | 3 |
|  | 100-300 | 2 | 3 |
|  | TOTAL | 76 | 100 |
|  |  |  |  |
| How many asymptomatic women would you estimate you receive from other units per year? | 0 | 53 | 68 |
|  | 1-9 | 8 | 10 |
|  | 10-19 | 8 | 10 |
|  | 20-49 | 6 | 8 |
|  | 50-99 | 1 | 1 |
|  | 100-200 | 2 | 3 |
|  | TOTAL | 78 | 100 |

## Screening offered at preterm birth surveillance clinics

The screening offered at preterm birth surveillance clinics (or about to be offered at clinics due to open soon) can be seen in Table 3 S2 below. Nearly all sites (97%) offer transvaginal cervical length measurements, most often undertaken by consultants (74%).

Table 3 S2 Screening offered at preterm birth surveillance clinics

|  | **Screening offered at preterm birth surveillance clinics** | n | % |
| --- | --- | --- | --- |
| What screening tests do you offer asymptomatic women at risk of preterm birth? (non-exclusive) | Transvaginal cervical length measurement | 84 | 97 |
|  | Qualitative fetal fibronectin | 3 | 3 |
|  | Quantitative fetal fibronectin | 37 | 43 |
|  | Actim Partus | 3 | 3 |
|  | Partosure | 4 | 5 |
|  | QUiPP App | 43 | 49 |
|  | Urinalysis | 61 | 70 |
|  | High vaginal swab | 43 | 49 |
|  | Low vaginal swab | 35 | 40 |
|  | Endocervical swab | 6 | 7 |
|  | Other – ActiProm | 1 | 1 |
|  | TOTAL | 87 |  |
|  |  |  |  |
| If you undertake transvaginal cervical length scans, who performs them? (non-exclusive) | Consultant | 62 | 74 |
|  | Speciality trainee | 18 | 21 |
|  | Non-training grade clinical staff | 4 | 5 |
|  | Research staff | 7 | 8 |
|  | Midwife | 17 | 20 |
|  | Ultrasonographer | 40 | 48 |
|  | TOTAL | 84 |  |

## Treatment offered at a preterm birth surveillance clinic

While most clinics (55%) do not offer prophylactic vaginal progesterone on history alone without ultrasound surveillance (see Table 4 in main manuscript), some (29%) reported that sometimes they did. In the free text comments associated with this answer, 52% (11/21) said it depended on the woman’s wishes. Some (38%, 8/21) would offer it ‘depending on the woman’s history’, 10% (2/21) if it had been successful in a previous pregnancy, one site said if she declined cervical ultrasounds, and one site said if it had been prescribed elsewhere (e.g., through her GP or IVF clinic).

Most clinics (44%) offer cervical cerclage with a braided suture as the preferred primary treatment for a short cervical length. However, 13% chose the option of ‘other’. The free text comments associated with this answer, 27% (3/11) said their treatments depended on the clinical context. Some sites (36%, 4/11), offer the women either cerclage or progesterone, while one site said they offer either cerclage or the Arabin pessary. One site said they offer cerclage, progesterone or Arabin as policy, while two sites offer the same three options but only as part of research, such as the SUPPORT study. One comment mentioned how type of suture material for cerclage depended on clinician preference. Two comments, 18%, mentioned the C-STITCH trial, and how the results of this could dictate what type of suture material they use in future.

The second most popular choice for answering the question on preferred primary treatment for a short cervical length was ‘combination’ treatment (22%). Free text comments associated with choosing this answer found that the majority, 78% (14/18), stated that their combination preference is for a cerclage plus progesterone. One comment stated that their unit’s preference is for a combination of progesterone, with either a cerclage or Arabin pessary utilised too. The comments also highlighted how 28% (5/18) feel that the treatment combination would depend on history and the woman’s preference.

The questionnaire asked sites what their unit’s choice of a secondary treatment would be if the primary treatment for short cervical length failed, as a free text answer. An equal number of comments (23%, 16/69) said they would try progesterone, or would try (23%, 16/69) a vaginal cerclage. Some sites, 17% (12/69) said they would try whatever they had not already utilised (e.g., if tried cerclage before they would offer progesterone, and vice versa). Some (3%, 2/69) would try to re-do the cervical cerclage if it had cut through. Others (17%, 12/69) discussed referring the woman to a tertiary centre, and/or having a transabdominal suture inserted either this pregnancy or in the next pregnancy. Some (4%, 3/69) reported their secondary treatment would be to do nothing.

## Impact of preterm birth surveillance clinics

Sites were asked in a free text question if providing their preterm surveillance clinic had a direct impact or effect on other parts of their service. Several (29%, 20/68) said no, others (10%, 7/68) said it was too early to tell. Some (13%, 9/68) thought it standardised care, meaning women received more consistent advice and higher quality care. This was partly due to women being seen by specialist clinicians, and partly because the presence of a clinic increased general clinician awareness. Several sites (9%, 6/68) felt it reduced pressure on their standard antenatal clinic, and 9% (6/68) said it reduced ultrasonography department pressures due to a reduction in cervical length referrals. Meanwhile 4% (3/68) thought it increased theatre pressure as cerclages increased, while another 4% (3/68) felt implementation of the preterm clinic reduced their preterm birth rate overall. One site highlighted that it provided continuity to women.

## Caring for women who arrived in threatened preterm labour

Most sites, 69%, utilise quantitative fetal fibronectin to assess symptomatic women at risk of preterm labour (see Table 4 S2 below). Of the 6 sites who wrote a free text comment under the option ‘other’, 67% (4/6) said they would undertake a clinical and/or vaginal/speculum examination. One site said it depended on the time of day. For example, if a woman presented at night a transvaginal cervical length scan was unlikely. Another site was in the process of ordering fetal fibronectin.

Table 4 S2 Caring for women who arrive in threatened preterm labour

|  | **Caring for symptomatic women who arrive in threatened preterm labour** | n | % |
| --- | --- | --- | --- |
| How do you assess symptomatic women at risk of preterm labour? (non-exclusive) | Transvaginal cervical length scan | 35 | 38 |
|  | Qualitative fetal fibronectin | 7 | 8 |
|  | Quantitative fetal fibronectin | 64 | 69 |
|  | Actim Partus | 15 | 16 |
|  | Partosure | 9 | 10 |
|  | QUiPP App | 47 | 51 |
|  | Other | 6 | 6 |
|  | TOTAL | 93 |  |
|  |  |  |  |
| If you selected the QUiPP App, is this: | Recommended in local guidance | 36 | 78% |
|  | Not recommended in local guidance but the majority use it | 7 | 15% |
|  | Not recommended in local guidance but some use it | 3 | 7% |
|  | TOTAL | 46 | 100 |
|  |  |  |  |
| What criteria would you usually use to decide to transfer a woman to another unit? | When her cervical length is less than ___ mm | 4 | 4 |
|  | Based on biomarker test result | 20 | 22 |
|  | Based on the QUiPP App result | 27 | 29 |
|  | Not sure | 3 | 3 |
|  | Other | 39 | 42 |
|  | TOTAL | 93 | 100 |
|  |  |  |  |
| For what indication would you offer tocoloysis? | Transfer to another unit | 6 | 6 |
|  | Steroids | 18 | 19 |
|  | Steroids AND transfer to another unit | 64 | 69 |
|  | Other | 4 | 4 |
|  | Don’t use | 1 | 1 |
|  | TOTAL | 93 | 100 |
|  |  |  |  |
| Do you offer ‘rescue’ cerclage for prolapsed membranes in asymptomatic women with no contraindications? | Yes | 49 | 52 |
|  | It depends on gestation | 31 | 33 |
|  | No | 12 | 13 |
|  | Only to recruit to research/trial | 2 | 2 |
|  | TOTAL | 94 | 100 |

Most sites, 42%, selected ‘other’ when asked their criteria to transfer a symptomatic woman to another unit. In the free text comments, 33% (13/39) said they do not or rarely transfer out as they were associated with a Level 3 neonatal unit, with 26% (10/39) saying that they would transfer a woman out if their Level 3 neonatal unit had capacity issues. A third of sites (33%, 13/39) would transfer out due to a woman’s gestation. The gestational age cut-off given varied from under 27 to under 32 weeks’ gestation, with the variation due to their site’s local neonatal unit level. Some, (15%, 6/39) said it depended on clinical judgement, 8% (3/39) said it depended on how dilated the woman’s cervix was, and one site would transfer if a woman had bulging membranes and required an emergency cerclage.

Four sites selected 'when cervical length is less than__mm' for their criteria to transfer a woman. From the free text comments, one site gave their cut off as 10mm, another gave 20mm and another gave 25mm.

Twenty sites selected that their criteria to transfer a woman was based on her biomarker result. From the free text comments, 55% (11/20) said this was based on it being a positive or negative result. Some (30%, 6/20) said it would be based on their fetal fibronectin result being higher than 50ng/ml, and 3 sites said it would be based on their fetal fibronectin result being higher than 200ng/ml.

Of the 27 sites who selected that their criteria to transfer a woman was based on the QUiPP app, 96% (26/27) said this would be when risk of delivery within 1 week is 5% of more on the app. One site (4%) said that this would be when her risk of delivery was calculated as being more than 5% within one week, and she was less than 28 weeks’ gestation.

The majority of sites (69%) offered tocolysis to give steroids and transfer the woman to another unit. Four sites selected the option ‘other’, with 75% (3/4) saying it depended on the woman’s individual circumstance, and one site not being sure.

The gestation from which sites said they normally offer steroids ranged from 21+6 – 28 weeks’ gestation for the lower bound, to 33 – 38+6 weeks’ gestation at the upper bound, in varying combinations. The most frequently noted gestation bounds were to offer steroids from 22 weeks’ gestation (36/90, 36%) or 23 weeks’ gestation (36/90, 36%), up until 34 weeks’ gestation (29/78 37%).
